# The Bergen 4-Day Treatment for Obsessive-Compulsive Disorder: Does It Work in a New Clinical Setting?

**DOI:** 10.3389/fpsyg.2019.01069

**Published:** 2019-05-17

**Authors:** Gunvor Launes, Inger Lill Laukvik, Tor Sunde, Ingrid Klovning, Kristen Hagen, Stian Solem, Lars-Göran Öst, Bjarne Hansen, Gerd Kvale

**Affiliations:** ^1^Sørlandet Sykehus, Kristiansand, Norway; ^2^Department of Clinical Psychology, University of Bergen, Bergen, Norway; ^3^OCD-Team, Haukeland University Hospital, Bergen, Norway; ^4^Department of Psychiatry, Molde Hospital, Molde, Norway; ^5^Department of Psychology, Norwegian University of Science and Technology, Trondheim, Norway; ^6^Department of Psychology, Stockholm University, Stockholm, Sweden

**Keywords:** obsessive-compulsive disorder, ERP, exposure therapy, B4DT, group therapy, outcome

## Abstract

Sørlandet Hospital in Norway has a history of offering patients with obsessive-compulsive disorder (OCD) cognitive behavior group therapy using 12 weekly sessions of 2.5 h each. A previous evaluation of this treatment has shown that 51.9% did not respond at post-treatment. Recently, a highly concentrated group-treatment format, the Bergen 4-day treatment (B4DT), has been shown to help more than 90% of patients with OCD post-treatment. Based on these positive results, it was decided to explore whether the B4DT could be a feasible format for delivering ERP at another clinic. Thirty-five consecutively recruited patients were included in the current pilot study, and assessed at pre-treatment, post-treatment, and 3-month follow-up. Treatment response rate (35% reduction in OCD-symptom score) was 94% at post-treatment, and 80% at follow-up. Seventy-four percent were in remission at post-treatment and 68% at follow-up. Only one patient dropped out of treatment. The patients were highly satisfied with the treatment content and format. The results indicate that the 4-day treatment could successfully be implemented at a new clinic.

## Introduction

Obsessive Compulsive Disorder (OCD) is a debilitating disorder that tends to become chronic when untreated (Skoog and Skoog, 1999). Since the late 1960’s, when Victor Meyer first published his work (Meyer, 1966), it has been established that exposure and response prevention (ERP) is an effective treatment approach that can be delivered in a number of formats (Olatunji et al., 2013), for example individually (Foa et al., 2005), in a group setting (Jonsson and Hougaard, 2009), spread over months (van Balkom et al., 1998) or concentrated (Hiss et al., 1994). When delivered in a group format (*d* = 0.24), the effect sizes are generally lower, as compared to individually (*d* = 0.50) delivered treatment (Öst et al., 2015).

Recently, results from a novel, ultra-concentrated format where the exposure-based treatment is delivered during four consecutive days, namely the Bergen 4-day treatment (B4DT), has been reported. The B4DT is delivered during four consecutive days to a group of 3–6 patients by the same number of therapists, and is often described as “individual treatment delivered in a group setting.” Following B4DT, 94% of the patients have responded (Havnen et al., 2014, 2017; Hansen et al., 2018a), and 68% were recovered at 1–4 years follow-up (Hansen et al., 2018a, 2019). In addition, the approach has shown significant effects on comorbid depressive symptoms as well as on generalized anxiety. The B4DT is highly accepted by the patients and there are basically no dropouts (Havnen et al., 2014, 2017; Hansen et al., 2018a; Kvale et al., 2018). These results can be compared with those achieved by standard ERP in the meta-analysis by Öst et al. (2015), where the response rate was 65% and the remission rate 50% (while often using a more lenient criterion than in the B4DT format studies above). Our own clinic’s previous work when using a 12-week group CBT approach showed that 51.9% did not respond at post-treatment (Håland et al., 2010), which was an important reason for the current pilot study with the B4DT.

The B4DT format was developed and introduced as part of standard care in an ordinary outpatient clinical setting with low selection of patients. The average duration of the OCD in the published effectiveness studies was 15.7 years (Hansen et al., 2019), and mean pre-treatment score on the Yale-Brown Obsessive Compulsive Scale (Y-BOCS; Goodman et al., 1989b), was 25.9 (Havnen et al., 2014, 2017; Hansen et al., 2018a). This makes the B4DT highly relevant for outpatient units offering treatment to patients with OCD.

Even though the B4DT is firmly rooted in ERP-treatment, the format is new, and a recent meta-analysis (Open Science Collaboration, 2015) of 100 replication studies in social-, personality-, and cognitive psychology found that only 36% of the studies replicated the results of the original studies, and the mean effect size decreased from 0.40 to 0.20. The promising clinical results seen following B4DT format makes it a highly relevant approach that might substantially influence the way treatment is delivered, if comparable results can be seen in new settings. This is the first replication study reporting on the results of the B4DT when delivered outside of the originators’ clinic to patients seeking help in the public specialist mental health care, and we expected to replicate the results from the previous published effectiveness studies.

## Materials and Methods

### Participants

In 2011, the Norwegian Health Authorities decided to establish 30 specialized OCD teams covering all health regions, with the aim of providing evidence-based treatment to all patients suffering from OCD (Kvale and Hansen, 2014). One of the main purposes was to bridge the gap between what is known as effective treatment for patients suffering from OCD, and what the patients actually receive (Shafran et al., 2009). In order to be able to monitor the effect of the treatment, all teams were required to use a standardized protocol for assessment and outcome measures. The current treatment was delivered by the specialized OCD team at Sørlandet Hospital (in the southern part of Norway) as part of ordinary care. On initiative from the hospital, the data collection, with the aim of summarizing the treatment outcome, was approved by the local data protection agency (“Personvernombudet” and “Norsk Samfunnsvitenskapelig Datatjeneste, NSD). When of interest for a broader audience than the hospital, results are encouraged by the hospital to be published in peer-reviewed journals.

The psychiatric outpatient clinic has a specialized team for treating patients with OCD. All patients with OCD from a catchment area of 290,000 are referred to this clinic, and if they fulfill the diagnostic criteria of OCD with a severity indicating need of treatment (score of 16 or more on Y-BOCS) they are offered treatment. It is, however, important to note that if patients are suicidal, psychotic, bipolar in an unstable phase, actively substance abusing or have an eating disorder with a BMI considered to be too low for initiating psychological treatment, these disorders have to be dealt with before psychological treatment for the OCD is initiated. All referred patients are assessed in a face-to-face interview by one of the therapists of the OCD-team. A total of 52 consecutively referred patients, with treatment demanding OCD, were referred to the clinic from October 2015 to September 2016. Two patients were suicidal, one suffered from an ongoing bipolar disorder, one had an ongoing drug abuse, and one suffered from an eating disorder that needed to be dealt with prior to any OCD-treatment. Six of the referred patients did not want any kind of exposure-based treatment. Among the 41 patients that were eligible for OCD-treatment, two patients were not fluent in Norwegian, and could due to this not participate in the B4DT, and one lived too far from the clinic to enable exposure in the home setting while participating in the group. These three patients were offered individual treatment.

Of the 38 patients who were offered participation in the B4DT, one did not want to participate in a group. Furthermore, one patient who was offered and wanted the B4DT moved to another part of the country before the treatment was initiated. Thus, 36 patients (10 men) were included in the B4DT groups. One patient dropped out on day 2 of the treatment, due to unwillingness to go on with exposure treatment, thus 35 of 36 patients completed the treatment. Twenty-five of these 36 were women (71%). The sample had a mean age of 30.43 (*SD* = 7.92). Thirty of the patients (85.7%) had received previous treatment. For details related to comorbidity and demographics, please refer to Table 1.

**Table 1:** Demographics and diagnostic characteristics of the sample (*N* = 35).

	*n (%)*		*N (%)*
Female gender	25 (71.4%)	Comorbidity (any disorder)	24 (68.6%)
Marital status		Depression	9 (25.7%)
Single	14 (40.0%)	Panic	8 (22.9%)
Cohabitant	21 (60.0%)	Social anxiety disorder	8 (22.9%)
Previous treatment		GAD	7 (20.0%)
Inpatient	6 (17.1%)	ADHD	4 (11.4%)
Outpatient	30 (85.7%)	PTSD	3 (8.6%)
Psychotropic drugs	14 (40.0%)	Bipolar-II	1 (2.9%)
Education		Specific phobia	1 (2.9%)
High-school	14 (40.0%)	Somatoform disorder	1 (2.9%)
College	21 (60.0%)		
Work status			
Working	13 (37.1%)		
Student	5 (14.3%)		
Not working/Sick leave	17 (48.6%)		


*Twelve of the 14 patients using psychotropic drugs used SSRIs, while two used hypnotics. GAD, generalized anxiety disorder; PTSD, post-traumatic stress disorder; ADHD, attention deficit hyperactivity disorder.*

### Primary and Secondary Outcome Measures

Primary treatment outcome was changes in Y-BOCS scores. Treatment response and remission were defined using a modification of the international consensus criteria (Mataix-Cols et al., 2016). Response is defined as a ≥35% reduction of the individual patient’s pre-treatment Y-BOCS score, and remission as the response criterion is fulfilled and the post-treatment Y-BOCS score is ≤12 points. Changes in OCD-severity were also measured by self-reports, as were secondary outcomes, namely symptoms of generalized anxiety and depression.

### Assessment

Patients were diagnosed at pre-treatment by an experienced OCD-therapist using the Mini-International Neuropsychiatric Interview (Sheehan et al., 1998). Before the B4DT groups, Y-BOCS (Goodman et al., 1989b) and the OCD-module in SCID-I for DSM-5 (First et al., 2015) were conducted by specially trained and independent assessors. The training of the assessors consisted of theoretical lessons as well as video demonstrations that illustrated different scores on the different items. This was followed by a rating of three videotapes where they had to demonstrate a minimum of 80% accuracy compared to an expert on 2/3 of the interviews in order to proceed. On SCID-I, a kappa-value of 0.80 and on Y-BOCS a maximum difference of ±2 points were employed. Then the assessor had to perform three live interviews with OCD-patients, which were videotaped and rated by a blinded expert (same requirements as above).

### Instruments

*Mini-International Neuropsychiatric Interview* (Sheehan et al., 1998) is a structured diagnostic psychiatric interview for DSM-IV and ICD-10. It has high concordance with other diagnostic instruments (Sheehan et al., 1998).

*Structural Clinical Interview for DSM Disorders* (SCID-5; First et al., 2015) covers Axis I psychiatric disorders according to DSM-5. SCID has high inter-rater reliability with Cohen’s kappa coefficients ranging 0.70–1.00.

*The Yale-Brown Obsessive Compulsive Scale* (Y-BOCS; Goodman et al., 1989b) interview version consists of 10 items covering the severity of obsessions and compulsions. The Y-BOCS has good psychometric properties (Goodman et al., 1989a,b). Scores range from 0 to 40 with higher scores indicating higher severity.

*The Dimensional Obsessive-Compulsive Scale-Short Form* (DOCS-SF; Eilertsen et al., 2017) is a 5-item self-report questionnaire similar in structure to the original DOCS. Patients indicate on a checklist if they experience any of the four presented obsessions (contamination, responsibility, unacceptable thoughts and symmetry/ordering, or “other”). The five items pertain to severity (0–8 scale) of their obsessions and compulsions during the last week. As with the Y-BOCS, DOCS-SF scores range from 0 to 40.

*The Obsessive-Compulsive Inventory Revised* (OCI-R; Foa et al., 2002) is an 18-item self-report inventory. OCI-R examines severity of obsessive-compulsive symptoms. Each item is rated on a 5-point Likert scale (0 = not at all, 4 = extremely), with a total score range of 0–72. The total score of the OCI-R provides information about the OCD severity, and there are subscales addressing the severity of different types of obsessions and compulsions: Washing, checking, obsessions, neutralizing, ordering and hoarding. The OCI-R has been shown to be a valid and reliable assessment tool (Foa et al., 2002; Solem et al., 2010).

*The Patient Health Questionnaire 9-item* (PHQ-9; Kroenke et al., 2001) is based on nine criteria for diagnosing depression in DSM-IV. Each item is rated on a four-point Likert scale (0 = not at all, 3 = almost every day), and the answers refer to the past 2 weeks. It has good reliability and validity (Kroenke et al., 2010), and suggested cut-off score for detecting major depressive disorder has varied from 8 to 11 in different studies (Manea et al., 2012).

*The Generalized Anxiety Disorder 7-item* (GAD-7; Spitzer et al., 2006) is based on the DSM-IV criteria for generalized anxiety disorder. Each item is reported on a four-point Likert scale (0 = not at all, 3 = almost every day), and the answers refer to the past 2 weeks. It has good reliability and validity (Spitzer et al., 2006), and suggested cut-off score for identifying GAD has varied from 7 to 10 in different studies (Plummer et al., 2016).

*Client Satisfaction Questionnaire 8* (CSQ-8; Nguyen et al., 1983) is an 8-item questionnaire which measures patient satisfaction with health services. Each item is scored from 1 (very low satisfaction) to 4 (very high satisfaction), and total score ranges from 8 to 32. The CSQ-8 has sound psychometric properties (Larsen et al., 1979; Nguyen et al., 1983).

### Treatment

Prior to the treatment, patients are given a detailed description of the outline of the treatment, and are asked to have no other appointments during the 4 days. A modified version of the Treatment expectancy questionnaire (Borkovec and Nau, 1972) is used, in which the patients rate on a 0–100 scale (a) How much the treatment approach makes sense, (b) Whether they would recommend it to a friend, (c) How likely it is that they will show up and engage fully in the treatment all 4 days, and (d) Whether they expect the treatment to have the desired effect. If they rate less than 70, this is taken as an opportunity to explore potential obstacles for successful treatment. They are also asked to prepare exposure tasks, and it is underscored that the exposures that the OCD likes the least, are often the most useful. Also, they are informed that it is most beneficial to start with the exposures that are likely to make the largest changes in their life. On the first day the group meets for approximately 4 h. The participants give a brief presentation of themselves, and group norms of how to best support each other are established. Then the patients as a group are given a presentation about OCD and how to do ERP. In the psychoeducation the importance of making fully use of the three coming days in order get the OCD out if their lives is emphasized. Also, the OCD is systematically externalized and described in negative terms as something craving attention and trying to trick them into ritualizing or avoiding. It is also underscored that their most important task is to clearly demonstrate that they are no longer willing to comply with the demands of the OCD. This also includes zero tolerance for rituals. All patients has in advance prepared suggestions for exposure tasks, and during the last part of day one, each patient presents their suggestions for the exposure tasks they think will initiate the largest change. Typically, exposure tasks, which combine different OCD-domains, are encouraged. The patients are informed that this should be their last day with OCD, and that they are not supposed to start ERP before the start of treatment on day two. Each day includes some serving of fruit, biscuits, coffee and tea, and day 2–4 patients and therapists share lunch, which is also combined with summarizing progress.

On the second day, the group meets for approximately 8 h. The day starts with a repetition of the principles for ERP, and the patients are then given a chance to review their own lists of exposure tasks from day one. The patients are introduced to the LET-technique, which implies that the patients are encouraged to do all exposures without any subtle avoidance, but instead lean into the anxiety. They are encouraged to increase uncertainty in situations where the OCD is demanding certainty. It is specifically underscored that they should start paying attention to exactly the moment when the OCD tempts them to start ritualizing, since this is the moment when they can choose either to comply with the OCD, or to do something incompatible with the urge to reduce anxiety, uncertainty and discomfort. Anxiety and discomfort are labeled the raw material for change and something to be searching for in order to be able to practice the LET-technique.

The patients are then split up into pairs with one certified 4-day therapist per patient. The therapists work as a team, which means that the patients may or may not be working with the same therapist the 2 days of exposures. The LET-technique is practiced in numerous relevant exposure tasks and settings with a therapist as a coach. In the middle of the day, the group meets for lunch and during the lunch the patients’ experiences are summarized. This session is directed by the group leader and follows a strict pattern where each patient share their progress with the group, specifically focusing on whether they are using the LET-technique fully. The patients rate their own performance on a scale from 1 to 6, where 6 indicates that they are leaning fully in. If the patients give themselves a score of 5 or less, this is taken as an opportunity for the group leader to explore the reasons for holding back, with the intention of helping the patient to identify obstacles that can be dealt with during the next session. The explicit goal for each patient is to perform all exposure tasks in a way where they will be able to give themselves a rating of 6. Since the leaning in technique is based on a voluntary decision and intentional act, this is a goal that is obtainable for all patients. This process is also very transparent, since as a minimum of one therapist has been observing and working with the patient. Anxiety is only focused in terms of whether they were able to “find gold,” and if not, the reasons are explored by the group leader. This session follows directly after lunch (often when the group is still eating) and typically last for 15–20 min.

During the second day, each patient should have worked through their most important exposure tasks, especially the tasks they anticipate to be most potent for creating change in their lives. If the patient is unwilling to do exposures in some specific and relevant settings, this is challenged by the therapist, and the patient is highly recommended and encouraged to fight all attempts and temptations from the OCD that in essence will prevent them from regaining a normal life.

The group meets again at approximately 3:30 pm to summarize and share their progress in the same manner as at lunch. Together with their therapists, the patients make an individually tailored plan for ERP for the evening, and these exposures are typically self-administrated. Each patient reports progress in at least one text message, typically sent to the therapist at 9 pm. In this message they focus on how much they are “leaning in” during the exposures on a scale from 1 to 6.

On the third day, the group meets for 8 h, with basically the same outline and structure as day 2. After lunch, the patients get the experience of employing the LET-technique without assistance from the therapist. In the afternoon, the patients are encouraged to invite relatives or important others for a lecture about OCD, ERP, focused on how to support the patients in their project of change. In addition, they have individualized exposure plans for the evening, and report progress to the therapists by text messages as the previous day.

On the fourth day, the group meets for approximately 4 h. The last day starts with a summary of the previous evening. The focus for the rest of the day is on how to maintain and continue the changes the patients have made in their lives, and they are taught principles for how to be their own therapist. Together with their therapist each participant makes a day-to-day plan for further exposure during the next 3 weeks. When they leave, they also get an appointment 3 months ahead for a follow-up session (30 min) which does not contain any ERP, but a repetition of the essential components of the treatment.

For the following 3 weeks, the patients are encouraged to report daily on how they are practicing the LET-technique. In this pilot study, it was done with paper-and-pencil and sent to the clinic once a week. The patients are informed that the clinicians read the reports, but that there would be no contact with the patients.

### Therapists

The treatment was delivered in nine groups by a team of five therapists; one psychiatrist and four psychologists. One of the nine groups had three patients, seven had four patients, and one group had five participants. The groups were allocated to pre-specified time slots, and patients were included upon availability. The therapists’ experience with OCD-treatment varied from 9 months to 15 years. Before the 4-day treatment was initiated, three of the five therapists received hands-on training in two B4DT groups led by the originators of the 4-day format, and two therapists were trained in one group. Thus, the originators participated as therapists in two of the 36 possible “therapist slots.” In the first two groups, therapists from the originators’ team assisted and evaluated the competency of the novel B4DT therapists.

### Adherence and Competency

The psychoeducation is highly manualized with a set of power point slides and accompanying text presented by the group leader. This is also the case for the psychoeducation delivered to the family. All participants (patients/family and therapists) have their own handouts, to increase transparency and adherence. As an important part of the format, brief therapist meetings are pre-scheduled throughout each of the 4 days, in order to enable adherence to the protocol, and also to give feedback to each therapist regarding the way the treatment is delivered. In addition, these meetings mean that all therapists are informed about the progress and challenges for each patient, which is a necessity in order to have more than one therapist working with a given patient, when considered useful. As a rule-of-thumb, the most experienced therapist works with the most challenging patient, and also assists and supervises juniors. The meetings also summarize how each therapist and patient work with the LET-intervention.

### Statistical Procedures

Repeated measures ANOVA were conducted to investigate change in symptoms from pre- to post- and follow-up as measured with Y-BOCS. Because the assumption of sphericity was not met, as assessed by Mauchly’s test of sphericity, χ^2^(2) = 7.65, *p* = 0.022, Greenhouse-Geisser correction was applied. The assumption of sphericity was not violated for the secondary outcome measures, therefore Wilks’ Lambda was used for these analyses. For post-hoc analysis, Bonferroni corrections were used. Effect sizes were calculated with Cohen’s *d*, defined as (Mpre-Mpost)/SDpre. In order to allow participants with missing data to be included in the analyses, missing data were replaced using the expectation maximization (EM) method of SPSS, version 24. When less than 25% of a data set is missing and the data are missing at random, which was clearly the case for this data set (Little’s MCAR test χ^2^(144) = 155.94, *p* = 0.234), EM is an efficient method of replacing data, as it requires no simulation of data sets (Schafer, 1997). There was relatively low amount of missing data (6.7% for Y-BOCS and 9.5% for the self-report measurements). We therefore chose the EM algorithm over multiple imputations, as it is suitable for conducting ANOVAs. For imputing the missing data, outcome variables at each time point were included (Schafer, 1997).

## Results

Results on primary and secondary treatment outcome measures are presented in Table 2.

**Table 2:** Means, standard deviations and effect sizes (Cohen’s *d*) for symptoms of OCD, anxiety, and depression.

	Pre	Post	Follow-up	*d* post	*d* F-U
Y-BOCS	26.74 (3.53)	10.87 (4.65)	9.97 (6.72)	4.50	4.75
DOCS-SF	26.09 (6.09)	–	11.52 (7.96)	–	2.39
OCI-R	25.24 (12.27)	–	8.80 (8.22)	–	1.34
GAD-7	12.02 (5.65)	–	5.79 (4.17)	–	1.10
PHQ-9	10.51 (6.05)	6.07 (5.99)	5.32 (4.46)	0.73	0.86


*Cohen’s *d*: Mpre – Mpost/SDpre. Y-BOCS, Yale-Brown Obsessive Compulsive Scale; DOCS-SF, Dimensional Obsessive-Compulsive Scale-Short Form; OCI-R, Obsessive Compulsive Inventory-Revised; PHQ-9, Patient Health Questionnaire-9; GAD-7, Generalized Anxiety Disorder-7.*

### Primary Outcome Measures

A total of 27 (77.1%) patients had a Y-BOCS score of 24 or higher (moderate to severe OCD) at pre-treatment. Only one patient had a Y-BOCS score above 16 at post-treatment. There were significant changes in OCD severity, *F*(1.657,56.342) = 159.95, *p* < 0.001, partial η^2^= 0.825. The change from pre-treatment to post-treatment was significant (*p* < 0.001), whereas the change from post-treatment to follow-up was not (*p* = 0.373). However, the change from pre- to follow-up assessment was significant (*p* < 0.001), indicating that the treatment effect was maintained from post-treatment to follow-up. The self-report measures included showed similar patterns. There were significant changes in OCD symptoms as measured with the DOCS-SF, *F*(1,34) = 107.15, *p* < 0.001, partial η^2^= 0.759, and the OCI-R, *F*(1,34) = 92.60, *p* < 0.001, partial η^2^= 0.731.

### Response and Remission Rates

Table 3 summarizes clinical improvement for the sample. Only two patients (5.7%) were unchanged at post-treatment whereas 33 (94.3%) showed a treatment response. A total of 26 patients (74.3%) were classified as in remission. At follow-up 20 % were unchanged and 80% showed a treatment response. A total of 24 patients (68.6%) were classified as in remission. Of the 26 patients who were in remission at post-treatment, four changed status to unchanged at follow-up, and two were classified as treatment responder. Of the seven patients in the response category at post-treatment, four went on to achieve remission, while one patient was classified at unchanged at follow-up. The two patients that were classified as unchanged at post-treatment were in the same category at follow-up. None of the patients were classified as deteriorated following treatment.

**Table 3:** Clinical improvement at post-treatment and follow-up.

		Status at follow-up, *N* (%)			
		Remission	Response	No change	Total
	Remission	20 (57.1)	2 (5.7)	4 (11.4)	26 (74.3)
	Response	4 (11.4)	2 (5.7)	1 (2.9)	7 (20.0)
Status post-treatment	No change	0 (0.0)	0 (0.0)	2 (5.7)	2 (5.7)
	Total	24 (68.6)	4 (11.4)	7 (20.0)	35 (100.0)


*Treatment response was calculated based on the international consensus criteria which requires a ≥35% reduction of the individual patient’s pre-treatment Y-BOCS score in order to be classified as having a clinically relevant treatment response. Patients classified as having a treatment response in Table 3 did not achieve remission. A patient is classified as remitted if the post-treatment Y-BOCS score is ≤12 points in addition to meeting criteria for treatment response. No patients reported deterioration at post-treatment.*

### Additional Comparisons

There were no significant post-treatment differences on Y-BOCS between patients with moderate and severe OCD at pre-treatment (Moderate: *M* = 8.56, *SD* = 2.03; Severe: *M* = 11.55, *SD* = 5.00), *t*(33) = 1.64, *p* = 0.11, or at follow-up (Moderate: *M* = 8.72, *SD* = 6.00; Severe: 10.34, *SD* = 6.98), *t*(33) = 0.59, *p* = 0.53.

A total of 75% of patients with moderate severity were remitted at follow-up compared to 66.7% of patients with severe-extreme OCD. A total of 91% of patients without comorbid disorders were in remission at follow-up compared to 58.3% for patients with comorbidity (Fisher’s exact test, *p* = 0.12), and the difference in Y-BOCS (Comorbidity: *M* = 11.45, *SD* = 7.03, No-Comorbidity: *M* = 7.74, *SD* = 4.83) was nearly significant, *t*(33) = 2.01, *p* = 0.053. Patients with psychotropic medication did not differ on Y-BOCS compared to patients without medication at pre-treatment, *t*(33) = 0.06, *p* = 0.95, post-treatment, *t*(33) = 0.43, *p* = 0.67, or follow-up, *t*(33) = 0.52, *p* = 0.61.

### Secondary Outcome Measures

For depressive symptoms (PHQ-9), Mauchly’s test was not significant (*p* = 0.119) and Wilks’ Lambda was used, *F*(2,33) = 15.24, *p* < 0.001, partial η^2^ = 0.480. The change from pre- to post-treatment was significant (*p* < 0.001), whereas the change from post-treatment to follow-up was not (*p* = 0.474). For depressive symptoms, 42.9% scored above the suggested cut-off value of 11 on PHQ-9 at pre-treatment compared to 11.6% at follow-up.

For symptoms of generalized anxiety (GAD-7), there were also significant reductions, *F*(1,34) = 44.56, *p* < 0.001, partial η^2^ = 0.567. At pre-treatment 65.7% scored above the suggested cut-off value of 10 compared to 20% at follow-up.

### Treatment Satisfaction

Patients were highly satisfied with the treatment, as indicated by a score of 29.5 (2.7) on the Client Satisfaction Questionnaire-8 where the maximum score is 32.

## Discussion

The aim of the current paper was to explore whether a concentrated exposure based treatment, the B4DT, would yield comparable results when delivered by new therapists at another clinic, and the results obtained are nearly identical with the results from the originators’ clinic. Treatment response rates for the current study was 94% at post-treatment and 80% at follow-up, while remission rates were 74% at post-treatment and 69% at follow-up. As comparison, in the previous studies on B4DT response rates range was 83–94% post-treatment and 76–91% at follow-up, whereas remission rate range was 74–77% post-treatment and 60–77% at follow-up. Also, the changes in self-reported depressive symptoms and in generalized anxiety were in line with what has been reported from previous effectiveness studies on the B4DT (Havnen et al., 2014, 2017; Hansen et al., 2018a). Furthermore, the patients expressed high satisfaction with all aspects of the B4DT format, including the amount of treatment. There was also a very low dropout rate (2.8%), which is approximately the same as previously reported with 1.3% in Hansen et al. (2019) and 0% in Hansen et al. (2018a).

Our clinic had substantial prior experience with group-based ERP, and compared to our previous results the B4DT was clearly superior. In our previous research (Håland et al., 2010), we applied substantially more liberal criteria for response (8 point reduction of pre-treatment Y-BOCS score), remission (a score of ≤14 and the response criterion). When applying these criteria on the current results, we find that only 2.9% of the patients who received the B4DT were unchanged following treatment compared to 51.9% of the patients in our previous study (Fisher’s Exact Probability test, *p* < 0.001). Also, in the current study with the B4DT 77.1% would have been classified as in remission, compared to 33.3% in our previous study (Fisher’s Exact Probability test, *p* < 0.001). Furthermore, only 14.3% of the patients who received the B4DT were unchanged at 3-month follow-up whereas the corresponding rate for our previous group treatments was 38.9%, which is also a significant difference (Fisher’s Exact Probability test, *p* = 0.02).

One obvious advantage of the B4DT, also compared to our previous 12-week intervention, is the ultra-concentrated format with clearly defined start- and endpoint that are agreed upon before the treatment is initiated. The fact that the patients ahead of treatment have allocated four full days to treatment, creates a highly change-focused atmosphere, since everyone knows that each minute matters. Due to the limited time, the patients were told that it is important to start working with the tasks that are likely to be associated with the largest change, and to combine as many different OCD-aspects during the exposures as possible. This clearly differs from our previous approach where the exposure followed a hierarchy, starting with tasks that were expected to elicit moderate discomfort, and then proceeding. Also, in the B4DT, disconfirmation of OCD-beliefs are actively replaced by the LET-intervention, where the ability to deal with uncertainty is the main purpose. Furthermore, the B4DT allows for individually tailored and therapist assisted exposure. The former approach only had two therapists treating 5–7 patients, and a substantial part of the exposures either were illustrated by engaging one or two patients, or were performed by the patients themselves without assistance from a therapist, most often as a homework assignments. Another important difference is the length of the sessions. Even though the B4DT can be considered ultra-concentrated, the two middle days actually can be seen as two prolonged exposure sessions, which facilitates both repetition of and variation in the therapist assisted exposures with the aim of making the change robust.

A major difference between the B4DT and our former approach addresses the attitude to anxiety and discomfort, where the B4DT regards the anxiety as the raw material (“gold to be digging for”) which is needed in order to obtain change as opposed to something to be avoided. Patients are taught be aware of all the micro-choices where the OCD tempts them to start to avoid or ritualize, and use these as opportunities to clearly show that they are choosing to do something incompatible with having OCD. By this, they learn a principle in dealing with uncomfortable emotions, thoughts and situations, which can be employed across settings. This approach to emotional regulation differs clearly from the approach in the former group program, where reduced anxiety and discomfort was seen as primary treatment goals.

Another important aspect of the B4DT format is the approach to training and adherence to the protocol, which are pinpointed in the important paper “Mind the gap: Improving the dissemination of CBT” by Shafran et al. (2009), as some of the major challenges that need to be dealt with effectively when disseminating treatments that work. Due to the 1:1 ratio between therapists and patients in groups of 3–6 patients, trainees can directly work with experts and receive feedback and supervision, while they simultaneously can observe 3–6 patients with OCD going through major changes during only 4 days. While such an approach with direct observation of experts combined with hands-on supervision is a cornerstone when training surgeons, the approach is very rare in psychiatry. Most therapists embrace the format and the opportunity it serves for learning, but for some therapists the format might be challenging, for example working with patients full days, sharing lunch with them, and also be observed directly by colleagues. In addition, the format requires the therapist to be mobile and flexible while at the same time be very focused. Furthermore, in order to be able to assist the patient, the therapists themselves need to regard anxiety and discomfort as gold and an opportunity for change as opposed to something dangerous, and to have tolerance for the patient’s discomfort and anxiety.

### Limitations

Despite the fact that some of the therapists from the originators’ site participated in the first two groups, this study can be seen as a successful step of systematic replication (Barlow et al., 2009), in which the disorder and primary outcome measure are the same but therapists and setting are new factors. However, the lack of a research design with a control condition obviously prevents firm conclusions related to causality, and a randomized controlled trial where the B4DT is compared to another treatment as well as to wait-list is warranted. Given the research design, we are not able to conclude that the B4DT is more effective than other treatments for OCD. Also, there are clear limitations related to which parts of the B4DT might be the most important for the clinical change observed. The study is also limited by the lack of long-term follow-up assessment. Future research should test if the B4DT works for other anxiety disorders, and a pilot study on panic disorder from our group has already shown promising results (Hansen et al., 2018b). Future research should also investigate the relationship between client satisfaction and treatment outcome.

## Conclusion

The main conclusion is that the B4DT format worked very well at the clinic, and the results are nearly identical to those reported from the originators’ clinic (Havnen et al., 2014, 2017; Hansen et al., 2018a, 2019). Following this pilot study, the B4DT is now the treatment of choice at our clinic, which also serves as a training site for new clinics.

## Ethics Statement

The treatment was delivered at Sørlandet Hospital, as part of ordinary care. This paper uses data collected as part of the standard assessment procedure at the outpatient OCD-clinic in Sørlandet Sykehus, Norway, approved by the Data Protection Official on September 28th, 2015. This treatment quality assurance strategy does not require full ethical review and approval according to national and institutional guidelines. This has been described by the Regional committees for medical and health research ethics in Norway (https://helseforskning.etikkom.no/reglerogrutiner/soknadsplikt/sokerikkerek?p_dim=34999&_ikbLanguageCode=us). Furthermore, quality assurance data does not require informed consent according to national and institutional guidelines. Since the data is quality assurance data as a part of the ordinary care, the patients did not sign an informed consent. The patients were informed that the data were collected as quality assurance data and their consent was implied through participation.

## Author Contributions

GL, GK, L-GÖ, and BH designed and planned the study. GL, TS, IL, IK, BH, and GK implemented the treatment. GL, GK, KH, L-GÖ, BH, and SS acquired, analyzed, and reviewed the data. GL, KH, SS, BH, and GK wrote, reviewed, and edited the manuscript. All authors edited and reviewed the manuscript.

## Conflict of Interest Statement

The authors declare that the research was conducted in the absence of any commercial or financial relationships that could be construed as a potential conflict of interest.
